# Autonomic instability, arrhythmia and visual impairment in a new presentation of *MTFMT* ‐related mitochondrial disease

**DOI:** 10.1002/jmd2.12355

**Published:** 2022-12-08

**Authors:** Caoimhe Howard, Arundhati Dev‐Borman, John Stokes, Declan O'Rourke, Ciara Gillespie, Eilish Twomey, Ina Knerr, Ritma Boruah

**Affiliations:** ^1^ National Centre for Inherited Metabolic Disorders Children's Health Ireland at Temple Street Dublin Republic of Ireland; ^2^ Bristol Eye Hospital University Hospitals Bristol and NHS Foundation Trust Bristol UK; ^3^ Department of Ophthalmology University Hospital Waterford Waterford Republic of Ireland; ^4^ Department of Neurology Children's Health Ireland at Temple Street Dublin Republic of Ireland; ^5^ Department of Radiology Children's Health Ireland at Temple Street Dublin Republic of Ireland; ^6^ School of Medicine University College Dublin Dublin Republic of Ireland

**Keywords:** autonomic instability, Leigh syndrome, MTFMT, optic atrophy

## Abstract

Mitochondrial methionyl‐tRNA formyltransferase (MTFMT) is required for the initiation of translation in mitochondria. Pathogenic variants in *MTFMT* have been described in association with clinical presentations with Leigh syndrome, as well with as multisystem involvement (particularly cardiac and ocular involvement). There is a spectrum of severity, but many reported presentations have been milder with a better prognosis than other pathogenic variants associated with Leigh syndrome. We describe the case of a 9‐year‐old boy homozygous for a pathogenic *MTFMT* variant (c.626C > T/p.Ser209Leu) who presented with hypertensive crisis on a background of hyperphagia and visual impairment. His clinical course was complicated by supraventricular tachycardia and severe autonomic instability, requiring intensive care unit admission. He also developed seizures, neurogenic bladder and bowel and had a markedly abnormal eye examination with bilateral optic atrophy. Magnetic resonance image brain showed abnormal high T2/fluid‐attenuated inversion recovery signal within the dorsal brainstem and in the right globus pallidus with some reduced diffusivity. Despite recovery from the acute neurological and cardiac manifestations, he has ongoing deficits in his gross motor skills and continues to have hyperphagia with rapid weight gain (approx. 20 kg in 2 years). Ophthalmic findings are persistent. This case expands the phenotype associated with *MTFMT* disease.

**Synopsis**

*MTFMT*‐related mitochondrial disease presents with multisystem involvement and can include less typical presentations with hyperphagia and autonomic dysregulation, in addition to more common neurologic, cardiac and ophthalmic presentations.

## 1 INTRODUCTION

The *MTFMT* gene at chromosome 15q22.31 encodes mitochondrial methionyl‐tRNA formyltransferase (MTFMT), which is required for the initiation of translation in mitochondria.^1^ While it has been shown that N‐formylation of the Met‐tRNA^Met^ is not an absolute requirement of mitochondrial protein synthesis, it appears to be crucial for the efficiency of synthesis, as well as respiratory chain complex stability and assembly into supercomplexes.^2^ Pathogenic variants in *MTFMT* have been associated with Combined Oxidative Phosphorylation Deficiency 15 (OMIM # 614947) and Mitochondrial Complex I Deficiency, Nuclear Type 27 (OMIM # 618248). Both are linked to clinical presentation with Leigh syndrome, in addition to multisystem involvement (particularly cardiac and ocular involvement).^3^, ^4^

Leigh syndrome is a progressive neurodegenerative mitochondrial disorder, which usually presents during the first year of life with neurological abnormalities such as hypotonia and movement abnormalities.^5^ Survival is poor, with death frequently occurring before 2.4 years.^6^ Case series of patients with *MTFMT*‐related Leigh syndrome have shown a milder phenotype and better prognosis than other genes associated with Leigh syndrome.^4^

We report the case of a 9‐year‐old boy who presented with subacute encephalopathy and marked autonomic instability, on a background of impaired vision and mild learning difficulties, who was found to have homozygous pathogenic variants in *MTFMT*.

## 2 CASE REPORT

A 9‐year‐old boy presented to hospital with a 3‐month history of increased appetite, fatigue and withdrawn behaviour, worsening in the preceding 3 weeks. The increased appetite was described as insatiable and was associated with corresponding weight gain (reported by parents, no documented weight prior to admission). His parents also reported sleep disturbance and headaches in the weeks prior. His parents were first cousins and members of the Irish Travelling Community. He had a history of mild learning difficulties and suspected developmental coordination disorder (a disorder of acquisition of and execution of coordinated motor skills), but had not been formally assessed. He also was under care of ophthalmology due to reduced vision, first detected at age 5 years, which was attributed to visual processing difficulties following normal ocular examinations and normal neuroimaging. However, his visual electrophysiology (pattern visual evoked potentials) at 6 years of age suggested visual pathway compromise, likely located at the level of the optic nerves. There was no known family history of note.

He was hypertensive at presentation with systolic blood pressure readings up to 183 mmHg, with associated tachycardia up to 175 beats per minute (BPM). The following day he developed a fever of 38.5°C without infective symptoms. Initial blood tests including full blood count, comprehensive metabolic panel, venous blood gas, creatine kinase, thyroid function, cortisol and preliminary infectious workup were within normal limits. Urine biogenic amine screen was normal. Electrocardiogram (ECG) showed sinus tachycardia and was concerning for left ventricular hypertrophy. Imaging including chest x‐ray and abdominal ultrasound was normal.

The patient was transferred to a tertiary paediatric hospital. On arrival, he was tachycardic with prolonged capillary refill time. Pulses were thready but regular. His abdomen was full with generalised tenderness and a palpable bladder. On neurological examination, he could not reliably follow instruction but moved all limbs symmetrically. Lower limb reflexes were brisk and plantar responses were downgoing. Echocardiogram at presentation showed moderate left ventricular (LV) dilatation and subjective evidence of reduced systolic function, but was limited. He subsequently developed concentric LV hypertrophy on repeat echo 3 days later with reduced ejection fraction (EF, 56%) and shortening fraction (SF, 26%), indicating LV systolic dysfunction.

**FIGURE 1:**
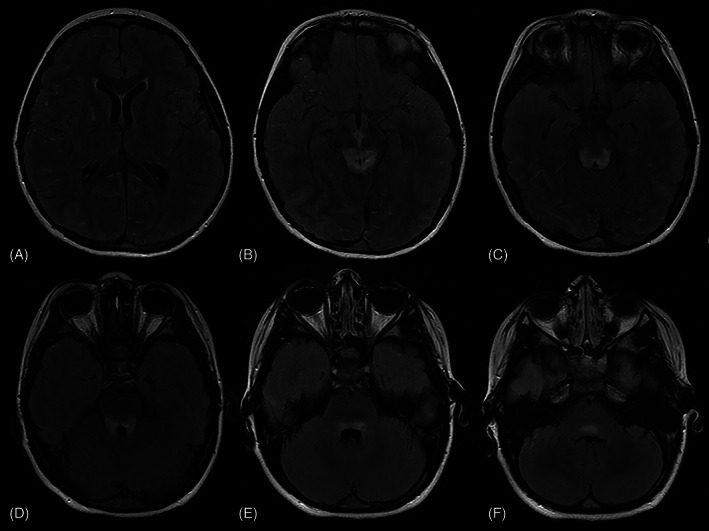
Magnetic resonance imaging of the brain on day 2 of admission showing T2/fluid‐attenuated inversion recovery (FLAIR) sequences. Imaging obtained at 9 years and 7 months of age. Axial T2/FLAIR sequences of the brain demonstrate T2/FLAIR hyperintensity within the right globus pallidus (A), dorsal mid brain and periaqueductal grey matter (B, C) and within the dorsal pons (D–F).

**FIGURE 2:**
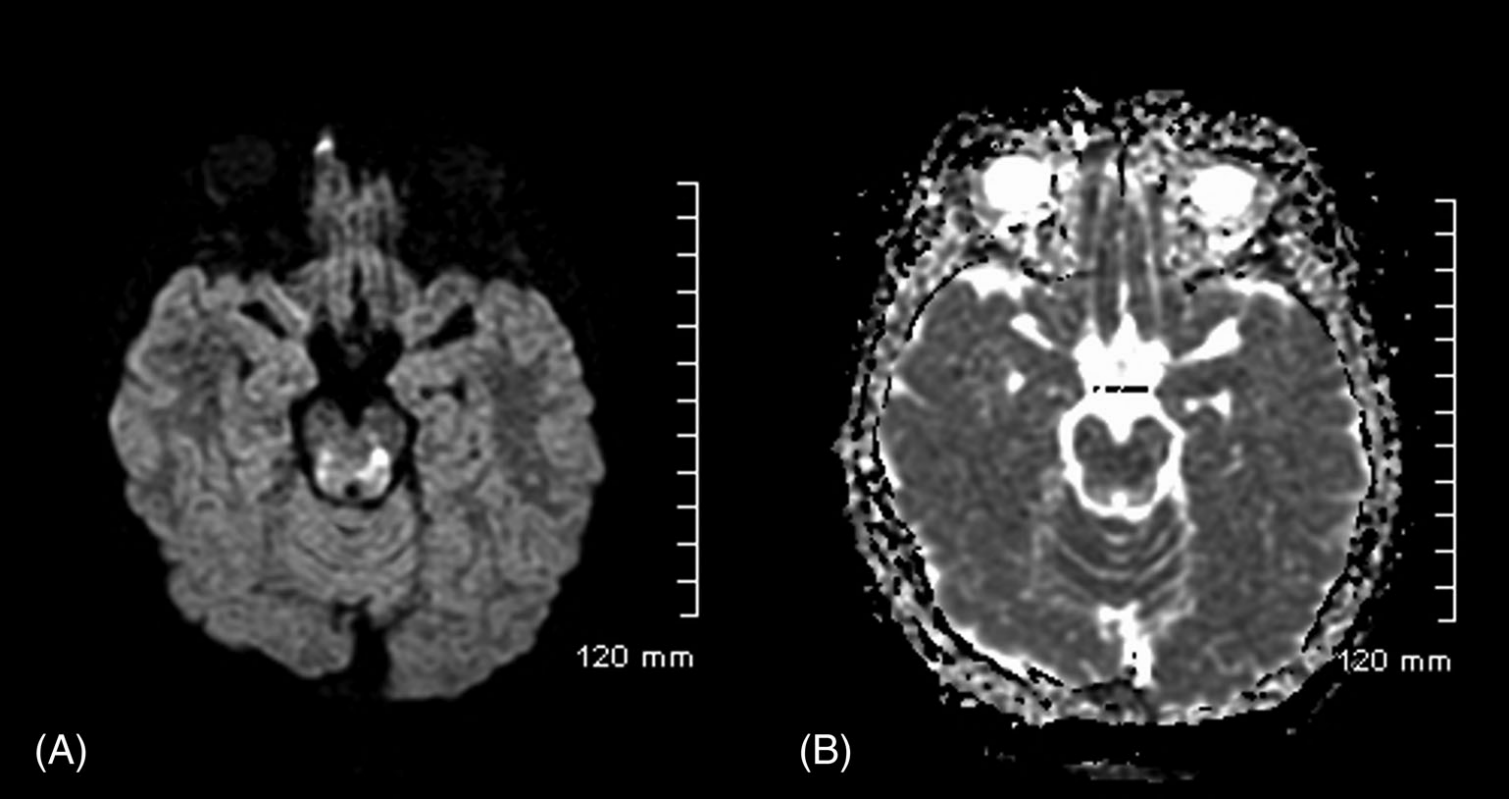
Magnetic resonance imaging of the brain on day 2 of admission showing diffusion‐weighted imaging (DWI) and apparent diffusion coefficient (ADC) mapping. Imaging obtained at 9 years and 7 months of age. Patchy reduced diffusivity seen within the midbrain on (A) axial DWI and (B) axial ADC map.

Computed tomography (CT) of the abdomen identified distention of the urinary bladder, requiring catheter insertion. CT brain showed a poorly circumscribed area of low attenuation centred on the tectal plate. Subsequent magnetic resonance imaging (MRI) was obtained with a GE Signa HD 1.5 Tesla scanner. Spinal MRI was normal. MRI of the brain identified abnormal high T2/fluid‐attenuated inversion recovery (FLAIR) signal within the dorsal brainstem, with reduced diffusivity in the left dorsal midbrain and faint areas of contrast enhancement. In addition, there was a small focus of high T2/FLAIR and low T1 signal in the right globus pallidus with no reduced diffusivity or contrast enhancement (Figures 1 and 2). The appearances were in keeping with a differential diagnosis of neuromyelitis optica, mitochondrial disease, or acute disseminated encephalomyelopathy (ADEM).

Cerebrospinal fluid (CSF) was obtained and infectious workup including viral investigations and cultures was negative. CSF cytology did not identify malignant cells. Following lumbar puncture, the patient was commenced on IV methylprednisolone due to the MRI appearances.

Initial metabolic screening is summarised in Table 1. An oral glucose load was performed and demonstrated a 30% post‐prandial increase in lactate from 2.33 to 3.05 mmol/L. Lactate/pyruvate ratio was normal at 13.3. He was commenced on coenzyme Q10, thiamine, riboflavin and biotin. Blood was sent for genetic testing, comprising of a next generation sequencing panel of nuclear genes associated with primary mitochondrial disorders, in addition to mitochondrial genome sequencing. Repeat MRI brain 5 days after the initial scan showed no significant interval change. MR spectroscopy with a 1.8 × 1.8 × 1.8 cm voxel placed on the left lentiform nucleus was also obtained and was normal.

**TABLE 1:** Summary of metabolic investigations at presentation

Biochemical investigations	Result	Reference range (if applicable)
Ammonia	25 μmol/L	0–65
Lactate	2.81 mmol/L	0–2.2
CSF lactate	2.41 mmol/L	1.1–2.2
Glucose	5.2 mmol/L	3.3–5.5
CK	50 U/L	20–155
Uric acid	229 μmol/l	100–350
Venous blood pH	7.44	7.28–7.40
Venous blood pCO_2_	4.0 kPa	5–7.2
Bicarbonate	21.7 mmol/L	20–27
Plasma amino acids (paired)	Normal	
CSF amino acids (paired)	Normal	
Acylcarnitine profile (dried blood spot)	Normal	
Urine organic acids (qualitative)	Mild increase in lactate excretion	

Abbreviations: CK, creatine kinase; CSF, cerebrospinal fluid.

During his intensive care unit (ICU) admission, the patient had multiple episodes of supraventricular tachycardia (SVT) requiring adenosine treatment. There was no ECG evidence of an accessory pathway. He was commenced on bisoprolol and then required addition of flecainide. Blood pressure was labile and he alternately required antihypertensive treatment and inotropic support during the admission. He remained in the ICU for a total of 15 days due to ongoing autonomic instability, seizures and fluctuating level of consciousness. Seizures were first noted on day 7 of the ICU admission and were myoclonic in nature. Electroencephalogram (EEG) demonstrated an abnormal slow background with no frank epileptiform discharges. He was commenced on levetiracetam with good effect. There were also ongoing concerns of impaired bulbar function and possible neurogenic bladder and bowel. He became polyuric but was able to maintain normal electrolytes. He gradually stabilised clinically and was able to transfer to the medical ward.

On the ward, he was able to increase his oral intake again to meet his requirements and his urine output normalised. He began to mobilise independently again with regular physiotherapy. A skin biopsy was taken and fatty acid oxidation flux in cultured fibroblasts excluded a primary fatty acid oxidation disorder, but did show reduced flux at 41°C (myristate oxidation 65% of controls, palmitate oxidation 42% of controls, oleate oxidation 31% of controls), consistent with the possibility of a secondary reduction in fatty acid oxidation such as a respiratory chain defect. Following review by cardiology day 23 of admission, flecainide and amlodipine were discontinued and he commenced atenolol with good response. Repeat echocardiogram on this occasion showed mild residual concentric LVH with EF (75%) and SF (43%) back within normal limits. Ophthalmic assessment at this stage identified bilateral reduction in visual acuity at, unaided, 6/36 right and 6/75 left eyes, respectively, with no improvement with pinhole. Colour vision was markedly reduced bilaterally. He had a reduced blink reflex with a staring, mask‐like face. His optic nerves were pale, indicating longstanding bilateral optic atrophy, which was in keeping with his poor visual function and electrophysiology findings.

The patient was fit for discharge on day 28 of his admission. On the day of his discharge, genetic results became available and revealed homozygous autosomal recessive variants in *MTFMT*. Homozygosity was confirmed with parental testing. The reported variant c.626C > T, p.(Ser209Leu), is a missense pathogenic variant, associated with Leigh syndrome and combined OXPHOS deficiency.

Since discharge, the patient has remained below baseline for gross motor function and requires ongoing physiotherapy. His ophthalmic findings are stable and he has been registered as sight impaired. He requires one‐to‐one help with his schooling due to his visual impairment and is under the care of the National Council for the Blind of Ireland. He had a normal repeat EEG and no further seizures therefore levetiracetam was discontinued several weeks after discharge. Echocardiograms at 9 months and 14 months post‐discharge were essentially normal. Blood pressure remained stable. He underwent Holter monitoring at 2 months and at 1 year after discharge and this was normal, so atenolol was weaned and discontinued. His recent brain MRI shows expected evolution of the prior lesions on the presenting scan and normal MR spectroscopy. His appetite is still increased and he continues to gain weight at a rapid rate (weight gain from 45.4 kg on the 98th percentile to 65 kg above the 99.6th centile in 22 months, with a height tracking on the 50th percentile). Dietary intervention is ongoing. In the absence of evidence of any long‐term clinical benefit thiamine, riboflavin and biotin were discontinued and he remains on monotherapy with coenzyme Q10, which his parents report does appear to be of benefit to his energy levels and visual function.

## 3 DISCUSSION

Our patient was homozygous for a known disease‐causing variant in *MTFMT*, c.626C > T, which is the most commonly reported pathogenic variant in the literature. Of the 42 previously reported patients, 34 (81%) are either compound heterozygous or homozygous for the c.626C > T variant. The variant results in two mRNA transcripts—one which produces p.Ser209Leu, and one which results in skipping of exon 4, causing a frameshift (p.Arg181Serfs*6) and premature termination of the protein.^1^, ^4^ The first cases of *MTFMT*‐related disease were reported in 2011 by Tucker et al., who demonstrated reduced MTFMT activity in the fibroblasts of two patients with Leigh syndrome, with reduced activity of complexes I and IV (and complex III in some) in patient cell lines.^1^ Since this report, there have been a number of cases reported, with the most comprehensive summary of cases to date (38 patients) published 2019 by Hayhurst et al.^4^ Haack et al. reported a minor allele frequency of about 0.1% in the European population for this common c.626C > T variant,^7^ suggesting that *MTFMT*‐related disease may be more frequent than is currently known.

There is a spectrum of disease severity reported with pathogenic *MTFMT* variants, ranging from a classical Leigh syndrome phenotype with early hypotonia, seizures, lactic acidosis and mortality in the first 2 years of life, to development of ocular or neurological symptoms in adolescence or adulthood.^4^, ^7^ The most common presenting features include developmental delay, gait abnormalities and ocular involvement, and around half of patients have some degree of cardiac involvement.^4^ Ocular abnormalities, affecting a third of patients in the series by Hayhurst et al., include, amongst others, nystagmus, strabismus, pigmentary retinopathy and optic atrophy.^4^ The majority of patients have elevated lactate on biochemical testing.

Our patient had a longstanding history of mild learning and coordination difficulties prior to his acute presentation, without evidence of developmental regression. While he did require some additional support for schooling, this was partially attributable to his poor vision, and he had never been impaired to the degree that further formal investigation had been instituted. This is in keeping with the milder spectrum of developmental issues reported in some patients. There does not appear to be a significant genotype/phenotype correlation. Our patient is homozygous for the c.626C > T variant, and of the other patients reported to have this genotype, two died before the age of 4,^4^ while one patient did not present until 44 years of age.^8^

Of the patients previously reported, 10 had required admission to the intensive care unit due to acute decompensation (55% due to respiratory complications).^4^ A number of these patients died during this admission, or were discharged home with long‐term ventilatory support. This is in contrast to our patient, who has done very well post ICU discharge and continues to make progress at home.

The subacute course preceding our patient's hospital admission included increased appetite and excessive weight gain. There was one patient previously reported with this phenotype,^9^ who was also hypertensive at presentation, but this patient was found to have elevated cortisol and ACTH in keeping with Cushing's syndrome, which our patient did not have. The lesions present on our patient's MRI brain included abnormal high T2/FLAIR signal within the dorsal brainstem. The regulation of food intake and energy expenditure is affected by a homeostatic control mechanism involving the hypothalamus, amygdala and brainstem. The dorsal raphe nucleus in the dorsal midbrain has been implicated in the regulation of food intake and has reciprocal connections with hypothalamic nuclei known to regulate feeding.^10^ This may be an explanation for the dysregulation observed in our patient.

Structural cardiac abnormalities are the most commonly observed abnormalities with *MTFMT* variants, including cardiomyopathy, septal defects and valve abnormalities. Five patients presented with an arrhythmia, with Wolff‐Parkinson‐White being the most common (three patients). Only one other patient has been reported with SVT without evidence of accessory pathway.^4^

Our patient had significant ocular involvement, with a history of impaired vision preceding his admission and longstanding bilateral optic atrophy. Optic atrophy, pigmentary retinopathy and ptosis are common ophthalmic features of mitochondrial diseases in general.^11^ Specifically, in the context of the phenotype associated with MTMFT variants, whilst optic nerve involvement is the most common feature, pigmentary retinopathy has also been reported.^4^ It is possible, therefore that, given his young age, our patient may still go on to develop further eye involvement, such as a pigmentary retinopathy, which may lead to further loss of vision. The boy remains clinically stable at over the past 2 years, but long‐term follow up will be required to fully characterise his clinical course.

Taken together, we here present previously unreported findings in a 9‐year‐old Irish boy, including autonomic instability and hyperphagia, in addition to previously described features including arrhythmia, optic atrophy, basal ganglia and brainstem lesions on brain imaging, and neurologic involvement with subacute encephalopathy, expanding the phenotypic spectrum of disorders associated with *MTFMT* variants.

## AUTHOR CONTRIBUTIONS

Caoimhe Howard reviewed the patient data and wrote the manuscript. Arundhati Dev‐Borman reviewed, co‐wrote the manuscript, provided details of the ophthalmic examination and outcomes and provided direct patient care. John Stokes reviewed the manuscript and provided ophthalmic care. Eilish Twomey and Ciara Gillespie reviewed the imaging and provided anonymised images for this publication and reviewed the manuscript. Declan O'Rourke co‐wrote the manuscript. Ina Knerr co‐wrote the manuscript. Ritma Boruah co‐wrote the manuscript and provided direct patient care.

## FUNDING INFORMATION

No funding was obtained for this study.

## CONFLICT OF INTEREST

The authors declare no conflicts of interest.

## ETHICS STATEMENT

All procedures followed were in accordance with the ethical standards of the responsible committee on human experimentation (institutional and national) and with the Helsinki Declaration of 1975, as revised in 2000 (5). Informed consent was obtained from all patients for being included in the study.

## Data Availability

Data archiving is not mandated but data will be made available on reasonable request.
